# Understanding social and clinical associations with unemployment for people with schizophrenia and bipolar disorders: large-scale health records study

**DOI:** 10.1007/s00127-024-02620-6

**Published:** 2024-02-20

**Authors:** Natasha Chilman, Dionne Laporte, Sarah Dorrington, Stephani L. Hatch, Craig Morgan, Celestin Okoroji, Robert Stewart, Jayati Das-Munshi

**Affiliations:** 1 Department of Psychological Medicine, King’s College London, Institute of Psychiatry, Psychology and Neuroscience (IoPPN), East Wing 3.16, De Crespigny Park, London, SE5 8AF UK; 2 King’s College London, ESRC Centre for Society and Mental Health, London, UK; 3 South London and Maudsley NHS Trust, London, UK; 4 NIHR Biomedical Research Centre, London, UK; 5 Health Service and Population Research Department, King’s College London, Institute of Psychiatry, Psychology and Neuroscience, London, UK; 6 Department of Psychological and Behavioural Science, London School of Economics, London, UK; 7 Black Thrive, London, UK

**Keywords:** Employment, Occupation, Schizophrenia, Bipolar disorder, Ethnicity, Natural language processing

## Abstract

**Purpose:**

People with severe mental illness (SMI) experience high levels of unemployment. We aimed to better understand the associations between clinical, social, and demographic inequality indicators and unemployment.

**Methods:**

Data were extracted from de-identified health records of people with SMI in contact with secondary mental health services in south London, UK. A Natural Language Processing text-mining application was applied to extract information on unemployment in the health records. Multivariable logistic regression was used to assess associations with unemployment, in people with SMI.

**Results:**

Records from 19,768 service users were used for analysis, 84.9% (*n* = 16,778) had experienced unemployment. In fully adjusted models, Black Caribbean and Black African service users were more likely to experience unemployment compared with White British service users (Black Caribbean: aOR 1.62, 95% CI 1.45–1.80; Black African: 1.32, 1.15–1.51). Although men were more likely to have experienced unemployment relative to women in unadjusted models (OR 1.36, 95% CI 1.26–1.47), differences were no longer apparent in the fully adjusted models (aOR 1.05, 95% CI 0.97–1.15). The presence of a non-affective (compared to affective) diagnosis (1.24, 1.13–1.35), comorbid substance use (2.02, 1.76–2.33), previous inpatient admissions (4.18, 3.71–4.70), longer inpatient stays (78 + days: 7.78, 6.34–9.54), and compulsory admissions (3.45, 3.04–3.92) were associated with unemployment, in fully adjusted models.

**Conclusion:**

People with SMI experience high levels of unemployment, and we found that unemployment was associated with several clinical and social factors. Interventions to address low employment may need to also address these broader inequalities.

**Supplementary Information:**

The online version contains supplementary material available at 10.1007/s00127-024-02620-6.

## Introduction

### Background

People who are diagnosed with schizophrenia and related disorders or bipolar disorders often experience significant disability and impairment. Unemployment rates remain high for those with these severe mental illnesses (SMI), estimated to reach between 80 and 90% for people with schizophrenia [1–5]. This has major implications for people with SMI on an individual level, as barriers to participating in the job market contribute to the social exclusion of people with SMI and can adversely impact quality of life and clinical outcomes [6]. In qualitative research, service users have highlighted the important role that employment can play in their lives and recovery [7, 8]. Unemployment is a sizeable contributor to the societal and economic costs of these mental illnesses, with lost productivity due to unemployment representing an estimated 29% of the total costs [5, 9].

Positive work histories and cognitive functioning are some of the significant predictors of employment outcomes for people with SMI [1, 10]. However, there is a relative dearth of information on associations between sociodemographic and clinical characteristics with unemployment outcomes for people with SMI. There is a need to assess these associations in SMI populations and to understand how these link to broader inequities impacting these groups, which could be used to inform intervention development and implementation.

The digitization of health records has allowed for large scale, naturalistic studies of the health of populations. However, studies using routine data to understand and assess inequalities in mental disorders have to date been hampered by a lack of individual-level measures of socioeconomic position, including employment and other indicators, as these are poorly or incompletely captured in standard structured fields in electronic health records (EHRs). In mental health EHRs, detail on the service user’s occupation may only be recorded in unstructured free-text fields in clinical note form [11], which limits its use to inform analyses, particularly in large-scale health records. However, text-mining methods can enable the extraction of this information. The present study utilizes this methodology to assess occupational status at scale in the health records of service users with SMI.

The aim of this study was to investigate sociodemographic, clinical, and service use variables associated with unemployment for service users with SMI. In addition, through using large-scale electronic health records from a diverse catchment area [12], we further sought to assess ethnic inequalities impacting this indicator. Based on the previous literature, we hypothesized that service users with SMI who had any recorded experience of unemployment would be more likely to be older [13–23], male [1], from a minority ethnic group [24], have a diagnosis of non-affective SMI [1], and have more intensive contact with mental health services as indicated by inpatient admissions [25, 26].

## Methods

### Participants and setting

The South London and Maudsley (SLaM) National Health Service (NHS) Trust serves a large, ethnically diverse catchment area across four inner-city London boroughs (Southwark, Croydon, Lambeth, and Lewisham), with a population of approximately 1.3 million people [27]. Compared with London as a whole, UK Census data indicate that there are higher proportions of people from Black African and Black Caribbean ethnic groups, and lower proportions from South Asian groups in the SLaM catchment areas [28]. Since 2008, the EHRs of service users have been de-identified and are accessible to approved researchers through the Clinical Record Interactive Search (CRIS) platform [27], which was accessed for this study.

**Fig. 1:**
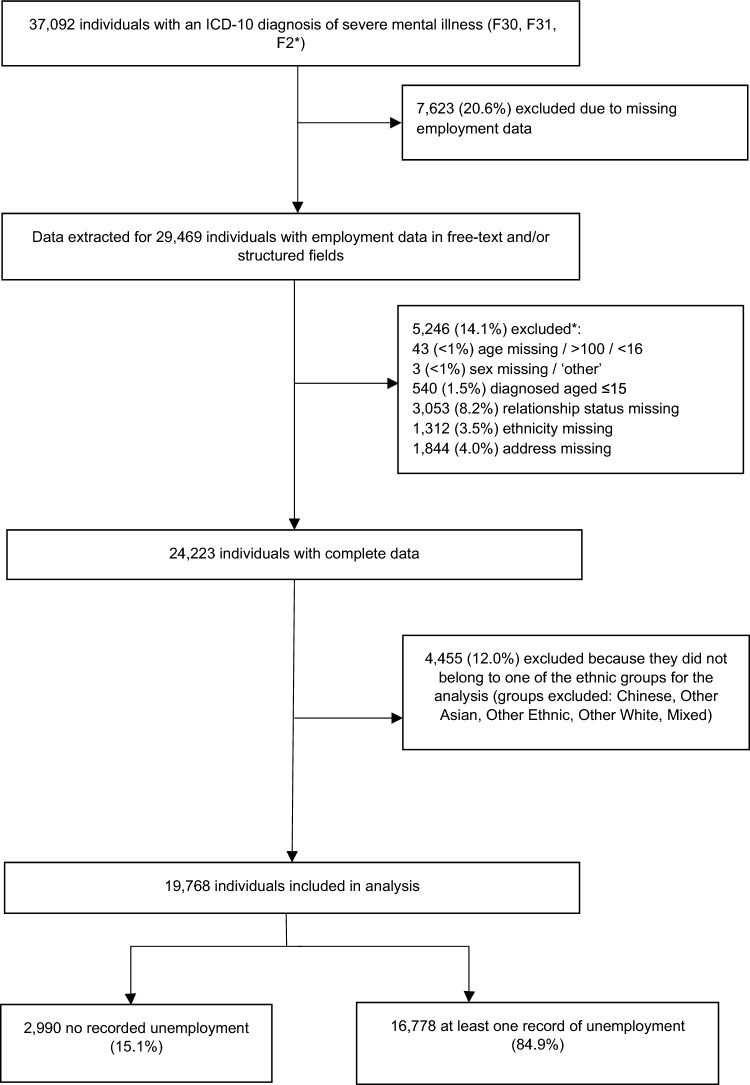
A flow-chart of the service users included and excluded from the sample, with total unemployment counts for the sample. *Groups are not mutually exclusive

SMI was defined as an F2* or F30/F31 disorder (schizophrenia and related disorders, and bipolar disorder) according to the International Classification of Diseases (ICD-10), where this diagnosis was not preceded by an F0* organic disorder (for example, dementia). Service user record IDs were extracted from CRIS where there had been a recording of a primary or secondary diagnosis of SMI, either in the structured field or the clinical notes in the record. The latter diagnoses were identified in the notes using an NLP application, developed using the Generalized Architecture for Text Engineering platform [29] to identify mental disorders according to ICD-10 diagnosis [30]. Service users were included if they were recognized as an adult in the UK (over the age of 16) at the time of occupation data extraction (29th January 2020), as this is the legal full-time working age in the UK. Sample sizes were small for inferential analyses in some of the ethnic groups (Fig. 1). Aggregating service users from these ethnic groups would have led to a heterogeneous group, which would be challenging to draw meaningful interpretations from. Therefore, similar to previous research, we excluded service users who did not belong to White British, Irish, Black African, Black Caribbean, Indian, Pakistani, or Bangladeshi ethnic groups [30].

### Measures

The service user’s date of birth was used to generate age at the time of data extraction. This was grouped into 10-year bands, except for the youngest group (age 16–29) to maintain sample power for this group with a start age of 16. Relationship status was grouped to include service users who were in a relationship (married/civil partnership/cohabiting), or not in a relationship (single/divorced/partnership dissolved/separated/widowed). Ethnicity was categorized to correspond with the Office for National Statistics categories for ethnicity [30]. We included service users who had ethnicity recorded as either White British, Irish, Black Caribbean, Black African, and South Asian (including Indian, Pakistani, Bangladeshi—these groups were aggregated due to smaller sample sizes). Area-level deprivation was assessed through the Index of Multiple Deprivation (IMD) [31], which is an aggregate of domains of area-level deprivation including income, employment, education, health, crime, barriers to housing and services, and living environment deprivation. IMD scores for the service user’s address closest to diagnosis date were mapped onto national quintiles of deprivation according to the Office for National Statistics 2015 guidelines related to Lower Super Output Area (LSOA), which have a mean of 1500 people residing in each area [32].

Several clinical and service use variables were extracted from the health record. Service users’ first SMI diagnosis (either from the structured field or clinical notes) was extracted and grouped into affective disorders (ICD-10 codes F30 and F31) and non-affective disorders (all F2* codes). The date of birth was used to calculate age at diagnosis date, which was grouped as working age SMI onset (< 64) or retirement age SMI onset (≥ 65). Service users who also had an ICD-10 diagnosis of F10–F19 were identified as having a history of alcohol or substance use disorder. We also extracted psychiatric inpatient admissions data which included: total number of inpatient admissions, total number of inpatient bed days, and total number of inpatient bed days under compulsory detention (the Mental Health Act). The median for cumulative bed days was calculated and used as a cut-off for the bed days groups.

### Employment status

To identify occupation mentions in the clinical notes in the EHR, we applied a validated text-mining application to extract occupation mentions from the clinical notes via CRIS [33]. The application uses a combination of machine-learning and rule-based approaches—the development of the application is described in further detail elsewhere [33]. The application has good levels of precision (0.88) and recall (0.90) on documents which describe the service user’s ‘personal history’ [33]. If the structured, drop-down field for employment was completed, this was also extracted. At least one mention of unemployment in either the clinical notes or structured field was used as an indicator of unemployment. Where a service user’s record had at least one mention of unemployment, they were included in the ‘unemployed group’. Where service users had multiple occupation types recorded in their records alongside unemployment (for example, ‘accountant’ and ‘unemployed’), they were also included in this ‘unemployed’ group. Conversely, if the record did not mention unemployment, the service user was included in the ‘never unemployed’ group. If occupation was not recorded in the health record (i.e., data were missing), these service user records were not included in the sample (Fig. 1). We validated this by manually checking the clinical notes for a random sub-sample of the cohort and comparing this to the extracted text-mined and structured field occupations. In this sample of 100 service users, this methodology gave a positive predictive value of 79% for the construct of interest (experience of unemployment) in manually screened full case notes.

### Statistical analyses

Stata-15 software [33] was used for all analyses. The characteristics of the sample were first cross tabulated with employment group. Univariable and multivariable logistic regression were used to assess associations between social, clinical, and service use characteristics and unemployment. A priori confounders included age and sex. We next included age, sex, area-level deprivation, relationship status, ethnicity, diagnosis type (affective/non-affective), age at diagnosis, and history of substance use disorder, as additional confounders in fully adjusted models. Likelihood ratio tests were conducted for all models. As an additional set of sensitivity analyses, we removed people above working age (65 +) from the sample and repeated the analyses.

## Results

Figure 1 displays the number of service user records identified with SMI (*n* = 37,092), and subsequent exclusion based on completeness of data and inclusion criteria. In total, 19,768 service users were included in the final analytical sample for complete case analysis (53.3% of original sample), of whom 16,778 (84.9%) had unemployment recorded.

Table 1 shows the descriptive data for service users in the complete sample. Most service users (85.3%) were recorded as not being in a relationship. While 51% of service users were White British, 28% of service users were Black Caribbean and 14% were Black African. A high proportion of service users were living in the most deprived areas at time of diagnosis (highest IMD quintile *n* = 8,978, 45%).

**Table 1:** Descriptive characteristics

	*N*	Percentage
**Total sample**	19,768	100
**Age**		
16–29	1986	10.0
30–39	3782	19.1
40–49	4143	21.0
50–59	4438	22.5
60–69	2634	13.3
70–79	1656	8.4
80–89	906	4.6
90–100	223	1.1
**Sex**		
Female	9419	47.6
Male	10,349	52.4
**Relationship status**		
In a relationship	2905	14.7
Not in a relationship	16,863	85.3
**Ethnicity**		
White British	10,102	51.1
Irish	606	3.1
Black Caribbean	5442	27.5
Black African	2809	14.2
South Asian	809	4.1
**Index for Multiple Deprivation (national quintiles)**		
1st (least deprived)	565	2.9
2nd	761	3.8
3rd	2428	12.3
4th	7036	35.6
5th (most deprived)	8978	45.4
**Diagnosis type**		
Affective	5573	28.2
Non-affective	14,195	71.8
**SMI onset**		
Late onset (65 +)	1907	9.6
Working age onset (under 65)	17,861	90.4
**Substance use disorder (ever)**		
No	16,289	82.4
Yes	3479	17.6
**Inpatient admission**		
No admissions	12,857	65.0
1 + admissions	6911	35.0
**Inpatient admissions: Bed days**		
No admissions	12,857	65.0
Low/moderate (< 78 days)	3446	17.4
High (≥ 78 days)	3465	17.5
**Compulsory admission**		
No detention	14,087	71.3
Detention	5,681	28.7

Table 2 shows the proportion of service users in the unemployed group for each sociodemographic group, and results from both the crude and adjusted multivariable logistic regression analyses. After adjusting for all covariates (age, sex, relationship status, ethnicity, diagnosis type, age at diagnosis, and substance use disorder), area-level deprivation was strongly associated with unemployment: service users who lived in the most deprived national quintile had twofold relative odds of experiencing unemployment compared with service users living in the least deprived areas. Service users had an increased odds of experiencing unemployment if they were middle aged at the time of data extraction, and not in a relationship. Although men were more likely to have experienced unemployment relative to women in unadjusted models (OR 1.36, 95% CI 1.26–1.47), differences were no longer apparent in the fully adjusted models (aOR 1.05, 95% CI 0.97–1.15).

**Table 2:** Sociodemographic associations with unemployment in service users diagnosed with severe mental illness

	N ever unemployed	Percentage ever unemployed	Unadjusted logistic regression^a^		Logistic regression adjusted for age and sex^a^		Logistic regression fully adjusted^a^^, b^	
			Odds ratio	95% CI	Odds ratio	95% CI	Odds ratio	95% CI
**Age**								
16–29	1528	76.9	Reference		Reference		Reference	
30–39	3260	86.2	1.87	1.63–2.15	1.85	1.61–2.13	1.84	1.60–2.12
40–49	3716	89.7	2.61	2.26–3.01	2.58	2.23–2.98	2.61	2.25–3.03
50–59	4034	90.9	2.99	2.59–3.46	2.97	2.57–3.44	3.01	2.59–3.51
60–69	2313	87.8	2.16	1.85–2.53	2.16	1.84–2.52	2.57	2.19–3.03
70–79	1254	75.7	0.94	0.80–1.09	0.94	0.81–1.10	2.17	1.76–2.68
80–89	554	61.2	0.47	0.40–0.56	0.49	0.41–0.58	1.80	1.36–2.37
90–100	119	53.3	0.34	0.26–0.46	0.36	0.27–0.48	1.39	0.97–2.00
**Sex**								
Female	7800	82.8	Reference		Reference		Reference^c^	
Male	8978	86.8	1.36	1.26–1.47	1.19	1.10–1.29	1.05	0.97–1.15
**Relationship status**								
In a relationship	2314	79.7	Reference		Reference		Reference	
Not in a relationship	14,464	85.8	1.53	1.39–1.70	1.59	1.44–1.77	1.33	1.19–1.49
**Ethnicity**								
White British	8231	81.5	Reference		Reference		Reference	
Irish	507	83.7	1.16	0.93–1.45	1.31	1.04–1.65	1.18	0.93–1.50
Black Caribbean	4883	89.7	1.99	1.80–2.20	1.92	1.73–2.13	1.62	1.45–1.80
Black African	2483	88.4	1.73	1.53–1.96	1.50	1.32–1.71	1.32	1.15–1.51
South Asian	674	83.3	1.13	0.94–1.37	1.11	0.91–1.35	1.17	0.95–1.43
**Index of multiple deprivation (national quintiles)**								
1 (least deprived)	394	69.7	Reference		Reference		Reference	
2nd	539	70.8	1.02	0.81–1.28	0.95	0.75–1.21	0.96	0.75–1.23
3rd	1922	79.2	1.63	1.34–1.99	1.50	1.22–1.85	1.29	1.04–1.61
4th	6022	85.6	2.47	2.06–2.98	2.22	1.83–2.69	1.81	1.48–2.22
5th (most deprived)	7901	88.0	3.08	2.57–3.70	2.75	2.27–3.32	2.12	1.73–2.60

^a^All likelihood ratio tests were significant (*p* < 0.0001) unless otherwise indicated

^b^Models were adjusted for age, sex, deprivation, relationship status, ethnicity, diagnosis type, SMI onset (late onset/ working age), and substance use disorder

^c^Likelihood ratio test for sex *p* = 0.2258

Across unadjusted and fully adjusted models for ethnicity, Black Caribbean service users had the highest odds of experiencing unemployment compared with White British service users (aOR 1.62, 95% CI 1.45–1.80). Black African service users were also more likely to have experienced unemployment compared with White British service users (aOR 1.30, 95% CI 1.14–1.49). There were no differences in the odds of unemployment between White British and Irish service users (aOR 1.18, 95% CI 0.93–1.50) or South Asian service users (aOR 1.17, 95% CI 0.95–1.43).

There were also associations between clinical and service use characteristics and unemployment (Table 3). In unadjusted models, service users had an increased odds of 1.43 (95% CI 1.32–1.55) of unemployment if they had a non-affective SMI diagnosis compared with an affective disorder. This association persisted after adjustment for all other covariates. The inpatient admission variables had the strongest associations with unemployment out of all variables: the odds of experiencing unemployment were elevated fourfold in service users that had experienced inpatient admissions in fully adjusted models. Service users who had inpatient admissions of a longer duration and compulsory admissions also had high odds of unemployment.

**Table 3:** Clinical and service use associations with unemployment in service users diagnosed with severe mental illness

	*N* ever unemployed	Percentage ever unemployed	Unadjusted logistic regression^a^		Logistic regression adjusted for age and sex^a^		Logistic regression fully adjusted^a^^, b^	
			Odds ratio	95% CI	Odds ratio	95% CI	Odds ratio	95% CI
**Diagnosis type**								
Affective	4537	81.4	Reference		Reference		Reference	
Non-affective	12,241	86.2	1.43	1.32–1.55	1.48	1.35–1.61	1.24	1.13–1.35
**SMI Onset**								
Late onset	1162	60.9	Reference		Reference		Reference	
Working age onset	15,616	87.4	4.46	4.03–4.94	3.52	2.84–4.36	3.44	2.77–4.27
**Substance use disorder (ever)**								
No	13,553	83.2	Reference		Reference		Reference	
Yes	3225	92.7	2.56	2.24–2.93	2.13	1.86–2.45	2.02	1.76–2.33
**Inpatient admission**								
No admissions	10,230	79.6	Reference		Reference		Reference	
1 + admissions	6548	94.8	4.63	4.13–5.19	4.65	4.14–5.23	4.18	3.71–4.70
**Inpatient admission(s) bed days**								
No admissions	10,230	79.6	Reference		Reference		Reference	
Low/moderate (< 78 days)	3187	92.5	3.16	2.76–3.61	3.05	2.66–3.50	2.78	2.42–3.19
High (≥ 78 days)	3361	97.0	8.30	6.80–10.13	8.68	7.09–10.62	7.78	6.34–9.54
**Compulsory inpatient admission(s)**								
No detention	11,402	80.9	Reference		Reference		Reference	
Detention	5376	94.6	4.15	3.67–4.69	4.01	3.54–4.55	3.45	3.03–3.92

^a^All likelihood ratio tests were significant (*p* < 0.0001)

^b^Models were adjusted for age, sex, deprivation, relationship status, ethnicity, diagnosis type, age at diagnosis, and substance use disorder

In the sensitivity analysis restricting the sample to working age adults only, no substantial differences were noted (Supplementary File 1).

## Discussion

In this large-scale sample of 19,768 service users with SMI, using novel text-mining methods, we found that an extremely high proportion (85%) of service users had experienced unemployment. Our findings are consistent with previous studies [2, 34]. Unemployment rates in the general population for the catchment area were estimated to be 4.3% in London by the Office for National Statistics at the time of the study [35], providing further context. Although these unemployment statistics for the local area capture unemployment at one particular timepoint, and in our study we looked at any recorded experiences of unemployment in the health record, our findings highlight the potentially deep inequalities which impact this group.

We also found key clinical differences between service users who had been unemployed compared with those who had no recorded unemployment. As hypothesized, service users who had experienced inpatient admissions, longer inpatient stays and compulsory admissions were more likely to have experienced unemployment. This can be considered in the context of previous work which has found that service users with SMI who have more severe symptoms and lower functioning scores are more likely to experience unemployment [36]. Our study provides a new perspective on this by investigating associations between inpatient stays and unemployment. An inpatient stay may suggest that the service user had more intensive contact with services and may have been more clinically unwell, which could have made it difficult to then initiate and maintain employment [36]. We also found that patients with comorbid substance use disorders had over twice the odds of experiencing unemployment. Substance use disorders were ascertained using ICD-10 codes; however, rates were lower than expected [37]. The presence of substance use disorders could therefore be an underestimate, due to under-recording in the structured field of the health record for some service users. In addition, service users with an earlier age of SMI onset and a non-affective SMI diagnosis were also more likely to experience unemployment in the sample—these could also be indicators of illness severity and functioning.

We found evidence to support our hypotheses for associations between sociodemographic characteristics and unemployment including age and relationship status, but not for sex. We found that service users who were aged between 50 and 59 were more likely to have experienced unemployment. These findings corroborate previous research: investigators have previously found that being of an older or middle age is associated with an increased likelihood of unemployment in people with SMI [13–23]. This may be a consequence of having more chances of being unemployed compared with younger service users. We found that service users who were not in a relationship were more likely to experience unemployment—this observation has been supported by work in other countries [38–42]. Although men had a higher likelihood of unemployment in the age adjusted model, this association was no longer evident in fully adjusted models, taking into account area deprivation, relationship status, ethnicity, and other clinical factors. Previous research on the relationship between sex and unemployment in SMI samples have also found no evidence of an association [17, 19–21, 25, 40, 42–48].

A key strength of the present study is that, using methodologies developed for large-scale textual analysis, we were able to assess a large sample of almost 20,000 service users. This electronic health record dataset provides ‘real-world’ data and insights [28] into occupation and unemployment for service users accessing mental health services in south London. As the study catchment area includes a high proportion of service users from Black African and Black Caribbean ethnic groups, we were able to undertake comparisons between minority ethnic subgroups, which has in general been limited and not usually possible to this extent. We hypothesized that there would be higher odds of unemployment for ethnic minority groups—this hypothesis was supported for some ethnic minority groups, but not others. Relative to White British service users, we found that Black Caribbean and Black African service users were more likely to have experienced unemployment, after adjusting for other variables, including area deprivation. Irish service users were also more likely to have experienced unemployment in age and sex adjusted models, although this was less apparent in fully adjusted models. We observed no differences between South Asian service users and White British service users in this sample. However, as Indian, Pakistani, and Bangladeshi ethnic groups were grouped as ‘South Asian’ due to smaller sample sizes, differences between these groups may have been masked, and it is a limitation that we could not disaggregate this group further. Overall, these findings suggest ethnic inequalities in employment outcomes for people with SMI are particularly apparent for Black Caribbean and Black African service users, but not Irish or South Asian service users.

Ethnic inequalities in unemployment are also evident in the general population in the UK: individuals from Black ethnic groups are currently twice as likely to be unemployed compared to White British individuals [49], and are more likely to be in precarious, temporary employment, which may exacerbate the risk of unemployment when unwell [50]. In addition, Black Caribbean, and Black African people in the general population have been shown to be more likely to receive lower earnings compared to White British people; these trends have also been noted in people of Pakistani and Bangladeshi origin [50]. A range of factors, including prior experiences of unemployment, younger age, lower education levels, and the impact of cumulative discrimination and racism over the life-course, have been implicated [50–52]. Our findings reflect these wider structural inequities, but with the additional focus on SMI impacting racially minoritized groups. Our findings are also consistent with other UK cohort studies: the investigators of the AESOP longitudinal study found that Black Caribbean people with first-episode psychosis were more likely to be unemployed compared with the White British reference group [24, 34].

A limitation of this study is that, by looking at mentions of ‘unemployment’, this may miss some service users who were unemployed where this was not mentioned in the health record. As unemployment was our primary outcome, we excluded patients who were missing occupation data from our sample. This recording and selection bias may impact some groups more than others. For example, older service users of retirement age may only be described as ‘retired’, despite experiencing unemployment prior to retirement age; therefore, unemployment may be disproportionately missed in this group. In a previous study, we found that people with more contacts with services, who may have a more severe course of illness, were more likely to have employment status recorded [33]. This may have partly influenced associations between unemployment and service use variables in the present study. However, our comparison group included service users who had no mentions of unemployment but did have other occupations, which could similarly be better recorded where service users had more contacts. Some other patient groups may be less likely to have occupation recorded and may therefore not be represented in the complete sample, for example those with communication difficulties, although this is likely to be a small number in the sample. Compared to a previous study with all secondary mental health service users, this group of patients with SMI had comparatively lower levels of missing employment data (20.6% in the present study, compared to 43.3% with all service users) [33].

We were unable to evaluate recall rates for the text-mining algorithms, as it was unfeasible to read through the service user’s whole care record when occupation is rarely mentioned in the context of the wider healthcare notes. However, our estimates of occupation recording after deploying the NLP application, which indicated that 79.4% of records had an occupation recorded (Fig. 1), approximated closely to the proportions previously reported in a national audit of SMI patients’ case notes [53]. Occupations should be well recorded for service users with SMI diagnoses, as employment history forms part of a holistic psychiatric assessment, with occupation support for people living with schizophrenia or bipolar disorders evidenced as a quality standard for delivering care [54]. As clinical notes tend to repeat information multiple times across the record [55], this increased opportunities for unemployment to be identified by the application. Furthermore, despite a risk that unemployment may have been under-recorded, our estimates of unemployment were still highly consistent with findings from other studies [1, 2, 56].

The present study was cross-sectional in design by extracting data held in the EHR in January 2020. It would have been desirable to look at patterns of unemployment longitudinally in service users with SMI—this was a key strength in the AESOP longitudinal study of outcomes for people with first-episode psychosis [56]. Limitations of the data and current methodology meant that we were unable to look at unemployment temporally in this study: we were unable to identify when a service user became unemployed, how long they were unemployed, or whether they were recently or currently unemployed. This further detail on unemployment would be of clinical and research interest. There were also other characteristics which could not be examined here—including service user’s educational attainment, and duration of untreated psychosis, which could have explained some of the variation in the analysis [13, 47, 57]. We were, however, able to measure other indicators of illness severity and functioning (inpatient admissions, length of inpatient stay, and compulsory admissions) and found that these indicators for more severe illness had strong and substantial associations with unemployment.

This study was conducted at a single secondary care mental health provider in an inner-city area in the UK, albeit with near-complete coverage of the catchment area of approximately 1.3 million people. The catchment area reflects an urban, ethnically diverse but highly deprived area in the UK [58]. The study catchment may be similar to other metropolitan/urban areas in the UK; however, the findings may be less generalizable to more rural catchment areas.

This study demonstrates that text-mining methodology can be useful to access and analyze the social determinants of mental health conditions in EHR data [59]. These approaches may be further developed in future to enhance understandings of inequalities in routinely collected health records data [60].

## Conclusions

The findings of this study make important contributions to the current literature by providing a large-scale assessment of the prevalence of unemployment experienced by secondary mental health service users with SMI. Our findings highlight the importance of a range of clinical and social indicators as impacting on the likelihood of service users experiencing unemployment. Our findings also suggest a potential ‘inequality within an inequality’ for service users from Black minority ethnic groups living with severe mental health conditions. Addressing high unemployment rates through support and interventions (for example, Individual Placement and Support [61]), which potentially take into account wider structural inequities, remains of paramount importance and could play an important role in future.

## Supplementary Information

Below is the link to the electronic supplementary material.

Supplementary file1 (DOC 94 KB)

Supplementary file2 (DOC 89 KB)

## Data Availability

Data are owned by a 3rd party SLaM BRC CRIS tool which provides access to anonymised data derived from SLaM electronic medical records. These data can only be accessed by permitted individuals from within a secure firewall (i.e. the data cannot be sent elsewhere) in the same manner as the authors.
