# Supplementary material for: Understanding social and clinical associations with unemployment for people with schizophrenia and bipolar disorders: large-scale health records study
Source: Soc Psychiatry Psychiatr Epidemiol. 2024 Feb 20;59(10):1709–19. doi: 10.1007/s00127-024-02620-6 (PMC11464607; doi:10.1007/s00127-024-02620-6)
Supplement: Supplementary file 1 — Supplementary file1 (DOC 94 KB) [file 127_2024_2620_MOESM1_ESM.doc]

**Supplemental File: Sensitivity analysis (sample restricted to working age adults)**

| **Table 1: Sociodemographic associations with unemployment in service users diagnosed with severe mental illness, in sample restricted to working-age adults only (aged between 16 and 64, n=16,150).** | | | | | | | | | | | |
| --- | --- | --- | --- | --- | --- | --- | --- | --- | --- | --- | --- |
|  | **N ever unemployed** | **Percentage ever unemployed** | **Unadjusted logistic regression^1^** | |  | **Logistic regression adjusted for age and sex^1^** | | |  | **Logistic regression fully adjusted^2^** | |
|  |  |  | **Odds Ratio** | **95% CI** |  | **Odds Ratio** | | **95% CI** |  | **Odds Ratio** | **95 % CI** |
| **Age:** | | | | | | | | | | | |
| 16-29 | 1,528 | 76.9 | Reference | |  | Reference | | |  | Reference | |
| 30-39 | 3,260 | 86.2 | 1.87 | 1.63-2.15 |  | 1.85 | 1.61-2.13 | |  | 1.85 | 1.60-2.13 |
| 40-49 | 3,716 | 89.7 | 2.61 | 2.26-3.01 |  | 2.58 | 2.23-2.98 | |  | 2.62 | 2.26-3.05 |
| 50-59 | 4,034 | 90.9 | 2.99 | 2.59-3.46 |  | 2.97 | 2.57-3.44 | |  | 3.02 | 2.59-3.52 |
| 60-64 | 1,607 | 89.2 | 2.48 | 2.07-2.97 |  | 2.47 | 2.06-2.97 | |  | 2.81 | 2.33-3.40 |
| **Sex:** | | | | | | | | | | | |
| Female | 7,841 | 88.8 | Reference | |  | Reference | | |  | Reference^3^ | |
| Male | 6,304 | 86.1 | 1.28 | 1.16-1.40 |  | 1.26 | 1.14-1.38 | |  | 1.04 | 0.94-1.15 |
| **Relationship status:** | | | | | | | | | | | |
| In a relationship | 1,798 | 84.1 | Reference | |  | Reference | | |  | Reference | |
| Not in a relationship | 12,347 | 88.1 | 1.39 | 1.23-1.58 |  | 1.64 | 1.44-1.87 | |  | 1.33 | 1.16-1.53 |
| **Ethnicity:** | | | | | | | | | | | |
| White British | 6,626 | 84.5 | Reference | |  | Reference | | |  | Reference | |
| Irish | 349 | 89.9 | 1.65 | 1.17-2.31 |  | 1.48 | 1.06-2.08 | |  | 1.28 | 0.91-1.80 |
| Black Caribbean | 4,323 | 91.7 | 2.02 | 1.79-2.28 |  | 2.06 | 1.82-2.32 | |  | 1.69 | 1.49-1.92 |
| Black African | 2,294 | 89.7 | 1.61 | 1.39-1.85 |  | 1.62 | 1.40-1.87 | |  | 1.37 | 1.19-1.60 |
| South Asian | 553 | 86.1 | 1.14 | 0.91-1.44 |  | 1.15 | 0.91-1.45 | |  | 1.21 | 0.96-1.54 |
| **Index of multiple deprivation (national quintiles):** | | | | | | | | | | | |
| 1 (least deprived) | 355 | 76.2 | Reference | |  | Reference | | |  | Reference | |
| 2^nd^ | 444 | 73.5 | 0.87 | 0.66-1.14 |  | 0.83 | 0.62-1.10 | |  | 0.87 | 0.65-1.17 |
| 3^rd^ | 1,655 | 82.5 | 1.48 | 1.16-1.88 |  | 1.42 | 1.11-1.81 | |  | 1.18 | 0.91-1.52 |
| 4^th^ | 5,002 | 88.1 | 2.31 | 1.84-2.90 |  | 2.12 | 1.68-2.67 | |  | 1.72 | 1.34-2.19 |
| 5^th^ (most deprived) | 6,689 | 90.5 | 2.96 | 2.36-3.72 |  | 2.69 | 2.13-3.38 | |  | 2.00 | 1.56-2.55 |
|  |  |  |  |  |  |  |  | |  |  |  |

^1^All likelihood ratio tests were significant (p<0.0001) unless otherwise indicated.
^2^Models were adjusted for age, sex, deprivation, relationship status, ethnicity, diagnosis type, SMI onset (late onset/ working age), and substance use disorder.
^3^Likelihood ratio test for sex p=0.4280

| **Table 2: Clinical and service use associations with unemployment in service users diagnosed with severe mental illness, in sample restricted to working-age adults only (aged between 16 and 64, n=16,150).** | | | | | | | | | | |
| --- | --- | --- | --- | --- | --- | --- | --- | --- | --- | --- |
|  | **N ever unemployed** | **Percentage ever unemployed** | **Unadjusted logistic regression^1^** | |  | **Logistic regression adjusted for age and sex^1^** | |  | **Logistic regression fully adjusted^2^** | |
|  |  |  | **Odds Ratio** | **95% CI** |  | **Odds Ratio** | **95% CI** |  | **Odds Ratio** | **95 % CI** |
| **Diagnosis type:** | | | | | | | | | | |
| Affective | 3,821 | 83.0 | Reference | |  | Reference | |  | Reference | |
| Non-affective | 10,324 | 89.4 | 1.73 | 1.58-1.90 |  | 1.67 | 1.51-1.85 |  | 1.39 | 1.25-1.55 |
| **Substance use disorder (ever):** | | | | | | | | | | |
| No | 11,102 | 86.1 | Reference | |  | Reference | |  | Reference | |
| Yes | 3,043 | 93.2 | 2.20 | 1.90-2.55 |  | 2.18 | 1.88-2.52 |  | 2.07 | 1.78-2.41 |
| **Inpatient admission:** | | | | | | | | | | |
| No admissions | 8,329 | 83.0 | Reference | |  | Reference | |  | Reference | |
| 1+ admissions | 5,816 | 95.1 | 3.94 | 3.47-4.47 |  | 4.47 | 3.93-5.09 |  | 3.91 | 3.43-4.47 |
| **Inpatient admission(s): bed days:** | | | | | | | | | | |
| No admissions | 8,329 | 83.0 | Reference | |  | Reference | |  | Reference | |
| Low/moderate (1-31 days) | 1,614 | 91.1 | 2.09 | 1.76-2.48 |  | 2.31 | 1.94-2.75 |  | 2.10 | 1.76-2.50 |
| High (32+ days) | 4,202 | 96.7 | 5.97 | 5.01-7.10 |  | 6.86 | 5.75-8.12 |  | 5.97 | 4.99-7.14 |
| **Compulsory inpatient admission(s):** | | | | | | | | | | |
| No detention | 9,321 | 84.3 | Reference | |  | Reference | |  | Reference | |
| Detention | 4,824 | 94.9 | 3.44 | 3.01-3.94 |  | 3.79 | 3.30-4.34 |  | 3.19 | 2.77-3.67 |

^1^All likelihood ratio tests were significant (p<0.0001) unless otherwise indicated.
^2^Models were adjusted for age, sex, deprivation, relationship status, ethnicity, diagnosis type, SMI onset (late onset/ working age), and substance use disorder.
